# Transcriptomic signatures of transfer cells in early developing nematode feeding cells of Arabidopsis focused on auxin and ethylene signaling

**DOI:** 10.3389/fpls.2014.00107

**Published:** 2014-03-24

**Authors:** Javier Cabrera, Marta Barcala, Carmen Fenoll, Carolina Escobar

**Affiliations:** Laboratory of Plant Physiology, Department of Environmental Sciences, Facultad de Ciencias Ambientales y Bioquímica, Universidad de Castilla-La ManchaToledo, Spain

**Keywords:** plant-nematode interaction, giant cells, early transcriptomic signatures, syncytia, auxin, ethylene, transfer cells, cell wall ingrowths

## Abstract

Phyto-endoparasitic nematodes induce specialized feeding cells (NFCs) in their hosts, termed syncytia and giant cells (GCs) for cyst and root-knot nematodes (RKNs), respectively. They differ in their ontogeny and global transcriptional signatures, but both develop cell wall ingrowths (CIs) to facilitate high rates of apoplastic/symplastic solute exchange showing transfer cell (TC) characteristics. Regulatory signals for TC differentiation are not still well-known. The two-component signaling system (2CS) and reactive oxygen species are proposed as inductors of TC identity, while, 2CSs-related genes are not major contributors to differential gene expression in early developing NFCs. Transcriptomic and functional studies have assigned a major role to auxin and ethylene as regulatory signals on early developing TCs. Genes encoding proteins with similar functions expressed in both early developing NFCs and typical TCs are putatively involved in upstream or downstream responses mediated by auxin and ethylene. Yet, no function directly associated to the TCs identity of NFCs, such as the formation of CIs is described for most of them. Thus, we reviewed similarities between transcriptional changes observed during the early stages of NFCs formation and those described during differentiation of TCs to hypothesize about putative signals leading to TC-like differentiation of NFCs with particular emphasis on auxin an ethylene.

## Introduction

Phyto-endoparasitic nematodes interact with their hosts in a subtle manner. They induce cells from the vascular cylinder to differentiate into specialized feeding cells (NFCs), syncytia, and giant cells (GCs), the nourishing cells for cyst and root-knot nematodes (RKNs), respectively (Jones and Goto, 2011; Sobczak and Golinowski, 2011). Although these two cell types clearly differ in their ontogeny, both develop cell wall ingrowths (CIs) believed to facilitate high rates of apoplastic/symplastic solute exchange typical of transfer cells (TCs; Jones and Dropkin, 1976; Siddique et al., 2012). In GCs the amplification of the plasma membrane surface area could be up to 20 fold (reviewed in Jones and Goto, 2011). CIs are less abundant in male-induced syncytia, suggesting a control probably associated with lower nutrient demand as the development of males inside the plant ends at the J3 stage (reviewed in Sobczak and Golinowski, 2009). Young syncytia are symplastically isolated, although this is lost at later stages (10–15 days post-infection, dpi; Hofmann et al., 2007) and symplastic isolation of GCs is under discussion (Hoth et al., 2008; Hofmann et al., 2010). Thus, amplification of plasma membrane area might be crucial for efficient apoplastic exchange at certain NFC developing stages. Polarized deposition of a thickened wall would need cell signals leading to this specialized differentiation. Those putative signals are still uncertain. Yet, some of the genes described to change their expression during TC differentiation participate in downstream cascades for gene expression driven by ethylene and auxin in epidermal cells of *Vicia faba* (Dibley et al., 2009) or in barley endosperm (Thiel et al., 2008, 2012a). In addition, genes involved in the biosynthesis of hormones such as auxin, ethylene jasmonic acid, brassinosteroids, gibberellins, and abscisic acid in maize basal endosperm (Xiong et al., 2011) are also differentially expressed in TCs.

Global transcriptomic changes of laser micro-dissected GCs at early developing stages (3 dpi; Barcala et al., 2010) and of micro-aspirated young syncytia (5 dpi; Szakasits et al., 2009) in Arabidopsis indicated that only 529 genes out of 1161 in GCs and 7225 in syncytia (representing 45.5 and 7.3% of the differentially expressed genes in each transcriptome, respectively) are shared. This indicates that transcriptomic similarities are not high between both nematode-induced cell types. However, key changes in the expression of genes related to several hormones such as auxin, ethylene, jasmonic acid, or abscisic acid are shared between both NFCs (Cabrera et al., 2013).

Only some data on TC regulatory signals in NFCs have been described, i.e., *ZmMRP-1* codes for a primary sensor of the putative signals for TCs and the activity of this TC-specific promoter *ZmMRP-1* (Gomez et al., 2002) was monitored in Arabidopsis transgenic plants; GUS activity was detected in the feeding sites induced by *Meloidogyne javanica*. Those are swollen parts of the roots (galls) where GCs are embedded surrounded by heterogeneous tissues. Although gall microscopy sections were not examined, the confined GUS activity observed suggests that the promoter is probably active in GCs (Barrero et al., 2009). Further research will elucidate whether this TC-specific molecular signature is also present in syncytia formed by cyst nematodes. In order to unveil some clues on putative signals leading to TC-like differentiation of NFCs, we review similarities between transcriptional changes observed during the early stages of NFCs formation and those described during differentiation of TCs. Global gene expression studies comparing syncytia and GCs to TCs signatures could be a starting point to find some answers.

## Auxin and ethylene as putative signals initiating TC-like morphology of NFCs

During the last few years, several transcriptomic studies on early developing TCs have assigned a major role to hormone regulation on the processes leading to the differentiation of these cell types. Auxin (Dibley et al., 2009) and ethylene (Thiel et al., 2008, 2012a,b; Dibley et al., 2009; Zhou et al., 2010; Xiong et al., 2011) raised as the two major phytohormones implicated in the differentiation of TCs from different plants and tissues. In adaxial epidermal cells of cultured *V. faba* cotyledons trans-differentiating to TCs, auxin and ethylene *cis*-responsive elements were over-represented in the promoters of induced genes. There is a clear functional demonstration that both hormones regulate CI formation in *V. faba* epidermis, based mainly in pharmacological experiments, indicating their participation in the signaling events leading to TCs differentiation (Dibley et al., 2009; Zhou et al., 2010). Moreover, an increase in auxin and ethylene levels boosts TC formation in tomato roots (Schikora and Schmidt, 2002). Accordingly, a clear over-representation of auxin-regulated genes among the 310 up-regulated genes in Arabidopsis 3 dpi GCs, relative to the total number of hormone responsive genes described in Nemhauser et al. (2006), was observed (Cabrera et al., 2013). Among those upstream genes regulated by auxin are genes in the *AUX/IAA* group, as *IAA8* (Table 1). Genes in this category were also induced in the transcriptome of *V. faba* developing TCs, such as *GH1* (Dibley et al., 2009). The development of *Heterodera shachtii*, a syncytia-forming nematode, was impaired in the *axr2* mutant, that also corresponds to the IAA group member *IAA7* (Goverse et al., 2000), and *IAA26* is induced in the transcriptome of 5 dpi microaspirated syncytia (Table 1; Szakasits et al., 2009). AUX/IAA proteins function by interacting with auxin responsive factors (ARFs) that were also induced in GCs, like *MONOPTEROS* (*MP; ARF5*) or *ARF19* (Table 1). In syncytia, *ARF4* and *ARF6* are up-regulated, but not co-regulated with GCs. *ARF4* has been defined to be preferentially expressed in the phloem companion cells of Arabidopsis (Table 1; Brady et al., 2007), the TCs found in loading and unloading areas of vascular tissues. Therefore, ARFs are induced in both NFCs triggered by cyst and RKNs at early infection stages, although different family members are involved in each NFC type. Strikingly, 20% of the genes defined by Brady et al. (2007) as preferentially expressed in the companion cells of Arabidopsis are also up-regulated in syncytia (44 out of 222 genes; Cabrera et al., 2013).

**Table 1:** **Genes induced in the 3 dpi GCs and 5 dpi syncytia transcriptomes (Szakasits et al., 2009; Barcala et al., 2010), that are also described to be regulated by auxin and/or ethylene in Nemhauser et al. (2006) and in Mapman (Thimm et al., 2004)**.

**Syncytia 5 dpi**			
**CELL**			
AT1G10740	IAA	1.0	Unknown protein
AT3G10530	IAA	2.3	Transducin family protein/WD-40 repeat family protein
**CELL WALL**			
AT1G53840	IAA	2.1	PME1: encodes a pectin methylesterase
AT2G39700	IAA	4.2	EXPA4: putative expansin. Naming convention from the Expansin Working Group
AT4G25810	IAA	5.1	XTR6: xyloglucan endotransglycosylase-related protein (XTR6)
AT5G06860	IAA	3.4	PGIP1: Encodes a polygalacturonase-inhibiting protein involved in defense response
AT5G57560	IAA	4.4	TCH4: Encodes a cell wall-modifying enzyme
AT5G66460	IAA	3.1	MAN7: (1-4)-beta-mannan endohydrolase
**DEVELOPMENT**			
AT5G57390	ACC	1.1	AIL5: Encodes a member of the AP2 family of transcriptional regulators
AT1G01470	IAA	2.0	LEA14: Encodes late-embryogenesis abundant protein
**HORMONE METABOLISM**			
AT1G48420	ACC	1.4	D-CDES: Encodes an enzyme that decomposes D-cysteine into pyruvate, H2S, and NH3
AT2G42680	ACC	1.1	MBF1A: One of three genes in *A. thaliana* encoding multiprotein-bridging factor 1
AT3G16050	ACC	2.0	PDX1.2: Encodes a protein with pyridoxal phosphate synthase activity
AT3G58680	ACC	1.6	MBF1B: One of three genes in *A. thaliana* encoding multiprotein-bridging factor 1
AT4G26200	ACC	1.2	ACS7: Member of a family of proteins in Arabidopsis that encode ACC synthase
AT4G34410	ACC	1.5	RRTF1: encodes a member of the ERF (ethylene response factor) subfamily B-3
AT5G20550	ACC	1.7	Oxidoreductase, 2OG-Fe(II) oxygenase family protein
AT5G43440	ACC	1.3	Encodes a protein whose sequence is similar to ACC oxidase
AT5G43450	ACC, IAA	1.2	Encodes a protein whose sequence is similar to ACC oxidase
AT1G17350	IAA	3.3	Auxin-induced-related/indole-3-acetic acid induced-related
AT1G50580	IAA	1.5	Glycosyltransferase family protein
AT1G56150	IAA	3.1	Auxin-responsive family protein
AT1G60690	IAA	0.9	Aldo/keto reductase family protein
AT2G02560	IAA	1.4	CAND1: *Arabidopsis thaliana* homolog of human CAND1
AT3G25290	IAA	1.6	Auxin-responsive family protein
AT3G25780	IAA	1.2	AOC3: Encodes allene oxide cyclase
AT3G30300	IAA	1.2	FUNCTIONS IN: molecular_function unknown; INVOLVED IN: biological_process unknown
AT3G63440	IAA	3.2	CKX6: encodes a protein whose sequence is similar to cytokinin oxidase/dehydrogenase
AT4G12410	IAA	2.8	Auxin-responsive family protein
AT4G12980	IAA	0.7	Auxin-responsive protein
AT4G27450	IAA	1.1	Unknown protein
AT4G34760	IAA	1.5	Auxin-responsive family protein
AT5G20810	IAA	0.4	Auxin-responsive protein
AT5G54510	IAA	2.7	DFL1: Encodes an IAA-amido synthase that conjugates Ala, Asp, Phe, and Trp to auxin
AT5G55540	IAA	0.8	TRN1: Encodes a large plant-specific protein of unknown function
AT5G64600	IAA	2.3	Unknown protein
**METAL HANDLING**			
AT2G37330	IAA	1.3	ALS3: Encodes an ABC transporter-like protein
**MISCELLANEA**			
AT2G29440	ACC	1.7	GSTU6: Encodes glutathione transferase belonging to the tau class of GSTs
AT3G11210	ACC, IAA	1.2	GDSL-motif lipase/hydrolase family protein
AT1G30760	IAA	6.2	FAD-binding domain-containing protein
AT2G30140	IAA	2.8	UGT87A2: UDP-glucoronosyl/UDP-glucosyl transferase family protein
AT3G11210	IAA	1.2	GDSL-motif lipase/hydrolase family protein
AT3G62720	IAA	1.3	XT1: Encodes a protein with xylosyltransferase activity
AT5G19440	IAA	2.3	Similar to *Eucalyptus gunnii* alcohol dehydrogenase
**N-METABOLISM**			
AT3G49640	IAA	1.5	FAD binding/catalytic/tRNA dihydrouridine synthase
**NOT ASSIGNED**			
AT3G02490	ACC	1.2	Pentatricopeptide (PPR) repeat-containing protein
AT4G33560	ACC	3.6	Unknown protein
AT5G49410	ACC	0.5	Unknown protein
AT5G02550	ACC, IAA	1.1	Unknown protein
AT1G03820	IAA	3.0	Unknown protein
AT1G08430	IAA	4.1	ALMT1: Encodes a Al-activated malate efflux transporter
AT1G18850	IAA	1.6	Unknown protein
AT1G28400	IAA	3.0	Unknown protein
AT1G32190	IAA	0.7	INVOLVED IN: N-terminal protein myristoylation; LOCATED IN: plasma membrane
AT1G32920	IAA	1.6	Unknown protein
AT1G55500	IAA	1.1	ECT4
AT2G34260	IAA	2.3	WDR55: transducin family protein/WD-40 repeat family protein
AT2G39725	IAA	2.5	Complex 1 family protein/LVR family protein
AT3G16310	IAA	2.4	Mitotic phosphoprotein N' end (MPPN) family protein
AT4G20170	IAA	1.5	GALS3
AT5G52910	IAA	0.6	ATIM: homolog of Drosophila timeless
AT5G64780	IAA	1.1	FUNCTIONS IN: molecular_function unknown; INVOLVED IN: biological_process unknown
AT5G66440	IAA	1.6	Unknown protein
**TRANSPORT**			
AT1G77380	ACC	2.7	AAP3: Amino acid permease which transports basic amino acids
AT2G39350	ACC, IAA	1.9	ABCG1: ABC transporter family protein
AT2G23150	IAA	3.2	NRAMP3: Encodes a member of the Nramp2 metal transporter family
**PROTEIN**			
AT1G26270	ACC	2.6	Phosphatidylinositol 3- and 4-kinase family protein
AT5G65450	ACC	1.1	UBP17: Encodes a ubiquitin-specific protease
AT5G63650	ACC, IAA	2.0	SNRK2.5: encodes a member of SNF1-related protein kinases
AT3G04230	IAA	1.8	40S ribosomal protein S16
AT3G17090	IAA	1.1	Protein phosphatase 2C family protein
AT3G60640	IAA	2.0	ATG8G: microtubule binding
AT4G22380	IAA	1.7	Ribosomal protein L7Ae/L30e/S12e/Gadd45 family protein
**REDOX**			
AT2G16060	ACC	1.3	HB1: Encodes a class 1 non-symbiotic hemoglobin
AT4G14965	IAA	1.0	MAPR4: heme binding
**RNA**			
AT1G21910	ACC	1.0	DREB26: member of the DREB subfamily A-5 of ERF/AP2 transcription factor family
AT1G71450	ACC	0.6	Member of the DREB subfamily A-4 of ERF/AP2 transcription factor family
AT1G77200	ACC	0.7	Member of the DREB subfamily A-4 of ERF/AP2 transcription factor family
AT3G16770	ACC	1.8	EBP: member of the ERF subfamily B-2 of the ERF/AP2 transcription factor family
AT5G43170	ACC	0.8	ZF3: Encodes zinc finger protein
AT5G67180	ACC	0.8	TOE3:|AP2 domain-containing transcription factor, putative
AT2G34140	ACC, IAA	0.7	Dof-type zinc finger domain-containing protein
AT1G16530	IAA	1.1	ASL9: Symbols: LBD3, ASL9 |ASL9 (ASYMMETRIC LEAVES 2 LIKE 9)
AT1G27730	IAA	1.2	STZ: Related to Cys2/His2-type zinc-finger proteins
AT1G30330	IAA	0.9	ARF6: Mediates auxin response via expression of auxin-regulated genes
AT2G33860	IAA	1.3	ARF3 (ETT) encodes a protein with homology to DNA-binding proteins which bind to AuxREs
AT2G47260	IAA	1.9	WRKY23: Encodes a member of WRKY Transcription Factor
AT3G11580	IAA	1.6	DNA-binding protein, putative
AT3G16500	IAA	1.8	IAA26 (PAP1) phytochrome-associated protein 1
AT3G23250	IAA	1.8	MYB15: Member of the R2R3 factor gene family
AT4G02220	IAA	1.2	Programmed cell death 2 C-terminal domain-containing protein
AT4G21550	IAA	1.4	VAL3: Symbols: VAL3 |VAL3 (VP1/ABI3-LIKE 3); transcription factor
AT5G60450	IAA	0.8	ARF4: member of the ARF family of transcription factors which mediate auxin responses
**SECONDARY METAB.**			
AT5G01210	ACC, IAA	2.1	Transferase family protein
**SIGNALING**			
AT1G35140	IAA	2.8	PHI-1
AT1G76650	IAA	1.3	CML38: calcium-binding EF hand family protein
AT2G25790	IAA	1.3	Leucine-rich repeat transmembrane protein kinase
AT2G30060	IAA	0.9	Ran-binding protein 1b
AT4G08950	IAA	3.8	EXO: EXORDIUM
AT4G28490	IAA	1.9	HAE: member of Receptor kinase-like protein family
AT5G05160	IAA	1.1	RUL1: leucine-rich repeat transmembrane protein kinase
AT5G12940	IAA	1.1	Leucine-rich repeat family protein
AT5G37770	IAA	2.0	TCH2: Encodes a protein with 40% similarity to calmodulin
**STRESS**			
AT2G42530	ACC	1.8	COR15B: COLD REGULATED 15B
AT5G64900	IAA	1.4	PROPEP1: Encodes a putative 92-aa protein that is the precursor of AtPep1
**Giant cells 3 dpi**			
**CELL WALL**			
AT4G25810	IAA	1.1	XTR6: xyloglucan endotransglycosylase-related protein
**HORMONE METABOLISM**			
AT3G23150	ACC	2.1	ETR2: Involved in ethylene perception in Arabidopsis
AT4G20880	ACC	2.4	Ethylene-responsive nuclear protein/ethylene-regulated nuclear protein
AT2G23170	IAA	1.0	GH3.3: IAA-amido synthase that conjugates Asp and other amino acids to auxin *in vitro*
AT3G50660	IAA	1.0	DWF4: hydroxylase whose reaction is a rate-limiting step in brassinosteroid biosynthetic pathway
AT4G27260	IAA	0.8	WES1: IAA-amido synthase that conjugates Asp and other amino acids to auxin *in vitro*
AT4G39400	IAA	1.4	BRI1: plasma membrane localized leucine-rich repeat receptor kinase
AT5G25190	IAA, ACC	3.2	ESE3: encodes a member of the ERF (ethylene response factor) subfamily B-6 of ERF/AP2 family
**LIPID METABOLISM**			
AT4G12110	IAA	1.9	SMO1-1: Encodes a member of the SMO1 family of sterol 4alpha-methyl oxidases
**METAL HANDLING**			
AT3G24450	IAA	1.8	Copper-binding family protein
**NOT ASSIGNED**			
AT4G33560	ACC	2.3	Unknown protein
AT2G39370	IAA	2.9	MAKR4: unknown protein
**POLYAMINE METABOLISM**			
AT5G19530	IAA	1.8	ACL5: Encodes a spermine synthase
**PROTEIN**			
AT3G61160	IAA	1.8	Shaggy-related protein kinase beta/ASK-beta (ASK2)
AT3G27580	IAA, ACC	2.5	ATPK7: a member of a subfamily of Ser/Thr PKs
**REDOX**			
AT2G16060	ACC	2.4	HB1: Encodes a class 1 non-symbiotic hemoglobin
**RNA**			
AT1G19220	IAA	1.6	ARF19: auxin response factor
AT1G19850	IAA	4.1	MP: Encodes a transcription factor (IAA24)
AT2G22670	IAA	1.2	IAA8: IAA8 (IAA8) gene is auxin inducible
AT3G02550	IAA	2.5	LBD41: LOB DOMAIN-CONTAINING PROTEIN 41
AT4G17460	IAA	4.3	HAT1: Encodes homeobox protein HAT1
AT4G36540	IAA	1.0	BEE2: BR Enhanced Expression 2
AT5G47370	IAA	2.7	HAT2: homeobox-leucine zipper genes induced by auxin
AT5G65310	IAA	1.1	HB5: class I HDZip (homeodomain-leucine zipper) protein
**SIGNALING**			
AT1G21980	IAA	1.4	PIP5K1: Type I phosphatidylinositol-4-phosphate 5-kinase
AT1G68400	IAA	1.8	Leucine-rich repeat transmembrane protein kinase
AT2G25790	IAA	1.1	Leucine-rich repeat transmembrane protein kinase

Third column shows the Log2 value for each gene in syncytia or GCs.

Other genes in the TCs induced in the epidermis of *V. faba* include those that could alter auxin transport through PINs relocalization or enhanced biosynthesis, as nitrilases (Dibley et al., 2009), proposed to alter the pattern of auxin redistribution to drive CI formation. Redistribution of PINs and reduction of nematode reproduction in *PIN*-related mutants such as *pin2*, *pin3*, *pin4*, *pin7*, and several double mutants, together with pharmacological treatments with inhibitors of auxin transport, demonstrated that cyst nematodes are able to hijack the auxin distribution network (Grunewald et al., 2009). Similarly, the nematode effectors Hg19C07 and Hs19C07 of *H. glycines* and *H. schachtii* directly interact with the auxin influx transporter LAX3 and infectivity in *aux1/lax3* double mutants was severely affected (Lee et al., 2011). Interpretations pointed as a process to facilitate the infection and establishment of nematodes during early stages of the plant-nematode interaction. However, the ability of CIs formation that lead to the TCs nature of NFCs, have not been ever studied in those mutants. Thus, further research with loss of function mutants will be needed to characterize auxin signaling pathways involved in TC character of syncytia and GCs.

Other downstream auxin responsive genes up-regulated in GCs are *WES1* and *GH3* (Table 1). In syncytia, there is also the *GH3*-like, *DFL1* that regulates lateral root formation through the auxin signaling pathway (Table 1; Nakazawa et al., 2001). The synthetic promoter *DR5*, derived from the *GH3* promoter, has been extensively used to check early increases in local auxin also in NFCs (Karczmarek et al., 2004). It is interesting to point that *DR5::GUS* expression is located in the GCs and vascular surrounding tissues (Cabrera et al., unpublished results). This suggests that auxin concentration is very high at early infection stages in GCs as compared to the rest of the gall tissues. There are several hypotheses to explain this local increase in auxin levels, i.e., the presence of auxins in the nematode secretions (De Meutter et al., 2005), or the manipulation of auxin homeostasis through the interaction of nematode effectors as chorismate mutase with the plant biochemical machinery (Jones et al., 2003). Interestingly, local production of isoflavonoids could also result in a local auxin increase as they inhibit polar auxin transport. Promoters of genes encoding chalcone synthases involved in the first step of flavonoid biosynthesis are induced in galls of clover (Grunewald et al., 2009), and genes in the category of flavonoid metabolism were induced in syncytia (Cabrera et al., 2013). However, several Arabidopsis mutants impaired in isoflavonoid biosynthesis were not affected in either syncytia or GCs formation (Jones et al., 2007; Wasson et al., 2009). These studies indicate that it is unlikely that flavonoids mediate changes in auxin transport needed for nematode feeding site organogenesis, although galls of *Medicago truncatula* flavonoid-deficient roots were shorter (Wasson et al., 2009). These findings suggest that the local flavonoid increase encountered in galls (Hutangura et al., 1999; Wasson et al., 2009) might have an alternative role not directly related to the process of infection and establishment. Perhaps flavonoids are related to CI formation needed for the acquisition of TCs characteristics, as suggested by Dibley et al. (2009) in TCs from epidermal cells of *V. faba*. This is another open question that deserves further investigation.

During differentiation of TCs in the endosperm of barley (Thiel et al., 2012a) or trans-differentiation of epidermal cells in *V. faba* (Dibley et al., 2009), ethylene is proposed to participate in a signaling pathway initiating TCs morphology. Similarly to the auxin responsive genes induced in the NFC transcriptomes, some genes involved in ethylene perception, transduction, and responses are also up-regulated in GCs (Table 1; *ETR2*, *ESE3*). Ethylene responsive genes were over-represented among the 310 up-regulated genes in GCs, relative to the total number of hormone responsive genes in Nemhauser et al. (2006), Cabrera et al. (2013). *ETR2* induction coincides with the presence of other ethylene receptors (as *ETR1*) in developing TCs from barley and rice endosperm (Thiel et al., 2012b). The ethylene precursor 1-aminocyclopropane-1-carboxylic acid (ACC) directly enhanced TC formation in root epidermal cells of tomato (Schikora and Schmidt, 2002) and adaxial epidermal cells of *V. faba* cotyledons (Dibley et al., 2009). Consistently, a pool of genes encoding proteins related with ethylene synthesis like two ACC oxidases (Table 1), are induced in the transcriptome of microaspirated syncytia. Functional analysis of the Arabidopsis ethylene overproducing mutants *eto2* and *eto3* resulted in hyper-susceptibility to cyst nematodes (Goverse et al., 2000; Wubben et al., 2001). Interestingly, ethylene overproduction in *eto2* mutants stimulated the formation of CIs or protuberances in syncytia along the vascular tissue, at late infection stages (Goverse et al., 2000), providing a direct evidence for a putative role of ethylene in the stimulation of syncytia TCs characteristics. Accordingly, functional analysis of Arabidopsis mutants compromised in several steps of the signaling cascade leading to activation of ethylene responsive genes, as those altered in ethylene-insensitive mutants (*etr1-1, ein2-1, ein3-1, eir1-1, and axr2*), were less susceptible to *H. schachtii* (Wubben et al., 2001). Hence, several *ACC synthase* coding genes were induced at early infection stages, increasing and reaching a maximum at 20 dpi in soybean infected with *H. glycines* (Tucker et al., 2010). Strikingly, ethylene production upon nematode infection has been long known in tomato infected with RKNs (*M. Javanica)*, with a peak at medium infection stages (4–16 dpi; Glazer et al., 1983), similar to several dicotyledonous species (Glazer et al., 1985). However, not much is known on the behavior of ethylene-related mutants infected with RKNs. Experiments on *Lotus japonica* expressing *ETR-1* were not conclusive of its putative role on RKN infection (Lohar and Bird, 2003). Undoubtedly, analysis are still lacking on the morphologic characteristics of developed syncytia in loss of function mutants of genes related to ethylene transduction pathways that could confirm their role on the induction of syncytia TCs characteristics, such us the presence of CI. Regarding RKNs, virtually no data on the TCs features of GCs in ethylene mutants are still available.

## Two-component signaling systems and reactive oxygen species as inducers of TC identity in NFCs

Recently, Thiel et al. (2012b) suggested a role for a two-component signaling system (2CS) in cellularization and differentiation of barley endosperm TCs, possibly coupled to hormonal regulation by abscisic acid and ethylene. In plants, 2CSs require a hybrid histidine kinase (HK; located in the plasma membrane) with both histidine kinase and receiver domains, a histidine-containing phosphotransfer protein (HPt), and a response regulator that mediates downstream signaling through phosphorylation. In Arabidopsis, proteins with significant sequence similarities to all elements of the 2CSs have been identified (reviewed in Schaller et al., 2008). We have searched for genes encoding those components in the early GCs and syncytia transcriptomes (Szakasits et al., 2009; Barcala et al., 2010; Table S1). In Arabidopsis, genes encoding 8 HK and 9 HK-like proteins (HKL) have been identified and 2 of them are up-regulated in GCs (*ETR2*) or syncytia (*PDK*). However, most HKs and HKLs are down-regulated in NFCs. *ETR2*, *HK2*, *HK3*, *ERS1*, and *PHYA* are down-regulated in syncytia and *HK1* and *CKI1* in GCs (Cabrera et al., 2013; Table S1). Moreover, the two up-regulated genes in NFCs are HKLs that lack residues essential to histidine kinase activity. The Arabidopsis genome encodes five HPt proteins (*AHP1-5*) that act as signaling intermediates between HKs and response regulators. From them, *AHP3* is up-regulated in syncytia while *AHP1* is down-regulated, and the rest of the genes are not differentially expressed in the transcriptome of micro-dissected GCs and syncytia (Barcala et al., 2010; Cabrera et al., 2013; Table S1). The last components of the system are the response regulators, with 33 genes identified in the Arabidopsis genome (23 response regulators and 10 pseudo-response regulators; Schaller et al., 2008), most of them down-regulated as well in NFCs (only *ARR7* and *RR14* are up-regulated in syncytia; Cabrera et al., 2013; Table S1). Thus, the transcriptomic evidence at early differentiation stages of syncytia and GCs suggests that genes involved in 2CSs do not contribute substantially to the differential gene expression observed in NFCs. Thus, 2CSs are not likely participating in the first signaling steps involved in the acquisition of TCs identify in NFCs.

Recently, it has been shown that H_2_O_2_ functions downstream of ethylene to activate cell wall biosynthesis and direct polarized deposition of a uniform wall on which CIs formed in TCs of *V. faba* cotyledons (Andriunas et al., 2012). The presence of a H_2_O_2_-generating mechanism dependent upon NADPH oxidase (NOX) activity was suggested (Andriunas et al., 2012). From the 10 *RBOH* genes encoding the catalytic subunit of NOX in Arabidopsis (reviewed in Sagi and Fluhr, 2006), 7 are down-regulated in syncytia, and none of them are differentially expressed in GCs (Cabrera et al., 2013). Additionally, defense-related genes described as related to TCs development, as in endosperm TCs where the ethylene response is possibly coupled to activated defense mechanisms (Thiel et al., 2008), are not abundant in NFCs. On the contrary, a general repression of plant defenses is obvious from the transcriptomes of early developing syncytia in Arabidopsis, where more than 35 peroxidases were repressed (Szakasits et al., 2009). Similarly, in early developing GCs of Arabidopsis and tomato not only peroxidases, but many genes from the secondary metabolism related to plant defenses were also down-regulated at 3 dpi (Barcala et al., 2010; Portillo et al., 2013). All these data suggest that active oxygen species such as H_2_O_2_ very unlikely function as early inducing signals for TCs identity of NFCs coupled to hormone signaling. However, it is important to point that we cannot discard the possibility of the activation of an oxidative burst in medium-late stages of NFCs development that might be participating in this process.

## Final remarks

It is interesting to point that many genes induced in early differentiating NFCs correspond to typical categories of downstream regulated genes that might participate in CIs formation, as those involved in vesicle trafficking, cell wall biogenesis, cell shape control and expansion, or nutrient transport, also induced in cells undergoing differentiation into TCs (Thiel et al., 2008, 2012a; Dibley et al., 2009; Xiong et al., 2011). Many other downstream genes are also induced in NFCs contributing to their development or maintenance (reviewed in Kyndt et al., 2013). Genes associated to changes in the cytoskeleton include those encoding tubulins, actins, microtubule-binding proteins, as the IAA-induced *ATG8G* (Table 1) or *AtFH6*, a formin encoding gene that regulates polarized growth by controlling the assembly of actin cables in Arabidopsis GCs (Favery et al., 2004). However, no functions directly associated to the TCs characteristics of NFCs have been yet described for those genes in NFCs (Kyndt et al., 2013). Other genes possibly related to active nutrient uptake into syncytia and GCs are also up-regulated by either auxin (*NRAMP3*), ethylene (*AAP3*), or both (*ABCG1*; Table 1). However, functional studies of these genes in NFCs are very scarce (reviewed in Kyndt et al., 2013). In addition, genes encoding cell wall modifying enzymes as pectin methylesterases, expansins, xyloglucan endotransglycosylases (*EXP4*, *XTR6*, *PME1*, *XTR6*), all induced by auxin, were also induced in NFCs (Table 1). Interestingly, the most clarifying study of a functional implication in TCs characteristic of NFCs, as the CIs formation, comes from the analysis of UDP-glucose dehydrogenase (UGD) coding genes. UGDs act through oxidation of UDP-glucose producing several cell wall polysaccharides. *UGD2* and *UGD3* are necessary for the production of CIs in syncytia and loss of function in double mutants severely affected nematode development (Siddique et al., 2012).

In conclusion, although genes encoding proteins with similar functions are differentially expressed in differentiating NFCs and typical TCs, a clear knowledge of their implication, either upstream or downstream, in the signaling cascades leading to TCs characteristics of NFCs is still lacking. Further research will probably elucidate the contribution of signals such as hormones to those differentiation events in NFCs.

## Conflict of interest statement

The authors declare that the research was conducted in the absence of any commercial or financial relationships that could be construed as a potential conflict of interest.
